# Linalool disrupts *Escherichia coli* biofilms via dual suppression of motility and adhesion

**DOI:** 10.3389/fvets.2025.1728048

**Published:** 2026-01-21

**Authors:** Lei Wang, Jingyan Zhang, Guowei Xu, Zhiting Guo, Jiamian Wang, Liping Huang, Lei Wei, Long Wang, Kang Zhang, Jianxi Li

**Affiliations:** 1 Traditional Chinese Veterinary Technology Innovation Center of Gansu Province, Key Laboratory of Veterinary Pharmaceutical Development of Ministry of Agriculture and Rural Affairs of China, Lanzhou Institute of Husbandry and Pharmaceutical Sciences of Chinese Academy of Agricultural Sciences, Lanzhou, China; 2 Zhengzhou Products Quality Inspection and Testing Center, Zhengzhou, China

**Keywords:** adhesion blockade, *Escherichia coli* biofilm, linalool, motility inhibition, natural antimicrobial agent

## Abstract

**Background:**

Bacterial biofilms, characterized by robust antibiotic resistance and involvement in chronic infections, present significant clinical challenges such as endometritis. While linalool as a natural extract exhibits potent antibiofilm properties, its precise mechanisms of action against *Escherichia coli* (*E. coli*) remain unclear.

**Methods:**

The inhibitory effect of linalool on *E. coli* biofilm formation was evaluated using inhibitory curve analysis and scanning electron microscopy. The influence of linalool on flagella and fimbriae formation in *E. coli* biofilms was assessed through swarming and swimming motility assays, scanning electron microscopy, and qRT-PCR. Viable count assays and confocal laser scanning microscopy were employed to examine the suppression of *E. coli* adhesion to bovine endometrial epithelial cells by linalool. Furthermore, an *in vivo* rat intrauterine infection model with *E. coli* biofilms was established to investigate the anti-adhesion activity of linalool.

**Results:**

*In vitro* assays demonstrated concentration-dependent biofilm inhibition by linalool, achieving 99% inhibition at 4 µL/mL, with structural disintegration confirmed through scanning electron microscopy. Mechanistically, linalool disrupted flagellar gene regulation, downregulating *fliA* and *motA* while upregulating *fliG* and *fliM*, and impaired both swarming and swimming motility. Simultaneously, it suppressed fimbriae-associated genes (*csgA, csgD*, and *fimH*), leading to 99% reduction in bacterial adhesion to bovine endometrial epithelial cells and the eradication of 95% of intrauterine biofilms *in vivo*.

**Discussion:**

As a low-toxicity phytochemical, linalool exhibits a dual-action mechanism in inhibiting *E. coli* D5 biofilm formation by suppressing motility and blocking adhesion, representing a potent multitarget agent against biofilm-associated infections. Future studies should validate its pharmacodynamics and potential synergies with conventional antibiotics to facilitate clinical application.

## 1 Introduction

Bacterial biofilms, structured microbial communities encased in extracellular polymeric substances, exhibit remarkable resistance to antibiotics and host immune defenses, contributing to persistent infections and therapeutic failures in clinical settings (1). *Escherichia coli* (*E. coli*), a prevalent pathogen, forms resilient biofilms linked to respiratory infections, digestive disorders, and reproductive system diseases, presenting significant therapeutic challenges (2, 3). Current approaches, including high-dose antibiotics administration and physical biofilm removal, often demonstrate limited efficacy and heightened risk of resistance development, emphasizing the urgent need for innovative therapeutic agents targeting biofilm-associated virulence mechanisms (4).

Natural products have reemerged as promising antibiofilm agents due to their multitarget potential and minimal risk of resistance development (5). Linalool, a monoterpene alcohol abundant in plant essential oils, exhibits broad-spectrum antimicrobial and anti-inflammatory properties (6–8). Recent studies have demonstrated its efficacy in inhibiting *Pseudomonas aeruginosa* biofilms by disrupting quorum sensing (9) and suppressing *Candida albicans* hyphal growth (10). However, its mechanism of action against *E. coli* biofilms, particularly concerning key virulence factors such as motility and adhesion, remains unexplored, presenting a critical gap in current knowledge.

Biofilm formation in *E. coli* is governed by two fundamental processes: (1) flagellum-driven motility, which facilitates surface colonization, and (2) fimbriae-mediated adhesion, essential for biofilm maturation (11). Flagellar assembly relies on key proteins such as FliG and FliM (rotor components) and MotA (stator complexes), while adhesion is mediated by curli and type I fimbriae, regulated by genes including *csgA*, *csgD*, and *fimH* (12, 13). Although phytochemicals such as cinnamomum and eugenol have been found to inhibit bacterial biofilm by inhibiting flagella protein synthesis and swarming motility or down-regulated fimbriae genes (14, 15), no compound has been reported to simultaneously disrupt both motility and adhesion in *E. coli* biofilms.

This study investigated the inhibitory mechanism of linalool on biofilms produced by the strong biofilm-forming strain *E. coli* D5 isolated from endometritis in dairy cows. Through a comprehensive approach integrating phenotypic assays, gene expression profiling, and *in vivo* models, linalool was demonstrated to disrupt biofilm formation via a dual mechanism: (1) dysregulation of flagellar gene networks, impairing motility, and (2) suppression of fimbriae biosynthesis, inhibiting bacterial adhesion. These findings not only elucidate linalool’s unique mode of action but also underscore its potential as a multitarget therapeutic agent against biofilm-associated infections, particularly within intrauterine environments.

## 2 Materials and methods

### 2.1 Bacterial strains and growth conditions

This study utilized *E. coli* D5, a strain isolated from the uterine mucus of Holstein cows diagnosed with clinical endometritis. Its accession number in GenBank is PX494327. This strain, characterized by its strong biofilm-forming capacity, was preserved at the Lanzhou Institute of Husbandry and Pharmaceutical Sciences, Chinese Academy of Agricultural Sciences (CAAS). A green fluorescent protein (GFP)-tagged *E. coli* D5 strain was also maintained at the same institute. Additionally, *E. coli* ATCC 25922, sourced from the American Type Culture Collection, was included in this study. Both strains were cultured in nutrient broth medium at 37 °C for 24 h before experimental application.

### 2.2 Crystal violet assay for *Escherichia coli* biofilm quantification

The antibiofilm efficacy of linalool against *E. coli* strains D5 and ATCC 25922 was evaluated using crystal violet staining, following the protocol described by Stepanovic et al. (16). Overnight cultures were adjusted to a concentration of 3 × 10^7^ CFU/mL in Luria-Bertani (LB) medium, and 100 μL aliquots were transferred to 96-well plates. Linalool (97%, Sigma) was added to an equal amount of dimethyl sulfoxide (DMSO) to mix well. Then the mixture was added to LB medium and mixed well, and then LB medium containing DMSO was used to dilute it in a two-fold manner to the required concentrations, maintaining the working concentration of DMSO in the solution at 1%. Linalool solutions (0.5, 1, 2, 4, 8, and 16 μL/mL) were added in equal volumes (100 μL per well), and incubation was conducted at 26 °C for 12 ~ 72 h. Following incubation, the medium was aspirated, and the wells were rinsed three times with phosphate-buffered saline (PBS, pH 7.4). After air-drying, biofilms were fixed in methanol (200 μL, 15 min), stained with 0.3% (w/v) crystal violet (200 μL, 5 min), and carefully washed. The bound stain was solubilized using 33% (v/v) glacial acetic acid (200 μL), and absorbance at 600 nm (n = 6 per group) was measured using the multi-mode reader (Synergy LX, BioTek, USA). Untreated bacterial suspensions served as controls, while sterile LB medium was used as a blank control.

### 2.3 Motility assays

The inhibitory effects of linalool on *E. coli* D5 motility were assessed through swarming and swimming assays. Swarming motility was evaluated following a modified protocol by Ranfaing et al. (17), whereas swimming motility was analyzed based on the method described by Li et al. (18) with slight modifications. The swarming medium contained 0.5% agar supplemented with 0.05% glucose, while the swimming medium consisted of 0.25% agar. Overnight cultures of *E. coli* D5 in LB medium were adjusted to a concentration of 4.5 × 10^8^ CFU/mL. Linalool (0.25, 0.5, 1, 2, and 4 μL/mL) was incorporated into autoclaved molten media at 50 °C before solidification. Plates were centrally inoculated with 0.2 μL of the bacterial suspension and incubated at 37 °C for 24 h. Motility inhibition was quantified by measuring the diameters of the migration zones extending from the inoculation points.

### 2.4 Cell culture and bacterial adhesion assay

Bovine endometrial epithelial cells, isolated and maintained at the Lanzhou Institute of Husbandry and Pharmaceutical Sciences, CAAS, were cultured in DMEM/F12 medium supplemented with 10% fetal bovine serum under a 5% carbon dioxide (CO_2_) atmosphere at 37 °C. Upon reaching 80% confluence, the cells were digested using 0.25% trypsin-ethylenediaminetetraacetic acid.

Adhesion assays were performed based on the methodology of Wultanska et al. (19), with modifications. Cells (2.5 × 10^5^ cells/mL) were seeded in 24-well plates and cultured until reaching 90% confluence. Following three PBS washes, wells were co-incubated with *E. coli* D5 (1.5 × 10^7^ CFU/mL) and linalool (0.5, 1, 2, and 4 μL/mL) for 2 h at 37 °C. Unattached bacteria were removed through two PBS washes. Adherent bacteria were lysed using 0.2% Triton X-100 for 10 min, serially diluted, plated on LB agar, and quantified after 24 h of incubation. Blank controls were included.

### 2.5 Confocal laser scanning microscopy (CLSM) for bacterial adhesion analysis

Bovine endometrial epithelial cells (5 × 10^5^ cells/mL) were seeded onto confocal-grade glass-bottom dishes and cultured at 37 °C with 5% CO_2_ until reaching 90% confluence. Following the aspiration of the culture medium and three PBS washes, the wells were incubated with GFP-tagged *E. coli* D5 (1.5 × 10^7^ CFU/mL) and linalool (0.5 and 1 μL/mL) for 6 h under standard culture conditions. Following the additional three PBS washes, the cells were stained with Hoechst 33342 for 15 min in the dark, followed by another three PBS washes. Imaging was performed using CLSM (LSM 700, Zeiss, Germany). Untreated control samples were also included in the experiment for comparison.

### 2.6 Rat intrauterine bacterial adhesion assay

This study was approved by the Lanzhou Institute of Husbandry and Pharmaceutical Sciences, CAAS (Approval No. 2022–017). *E. coli* D5 biofilms were cultured at 26 °C for 48 h, subjected to ultrasonication (20 Hz, 1 min), thoroughly mixed, and centrifuged (4,000 × *g*, 10 min) before resuspension in sterile saline (10^10^ CFU/mL). Nulliparous female SD rats (230 ± 20 g, 9 weeks old; n = 6 per group) were anesthetized with inhaled isoflurane (4% for induction and 1.5 ~ 2% for maintenance) used by an animal anesthesia ventilator (R660, RWD Life Science Co., Ltd.). Then the rats were inoculated intrauterine with 0.1 mL of biofilm suspension. Tail elevation maintained for 2 ~ 3 min to prevent reflux.

At 24 h post-inoculation, the experimental rats were treated with intrauterine administration of linalool (0.05 mL/100 g·BW) under inhaled isoflurane anesthesia. The groups were divided as following: (1) blank control (saline perfusion), (2) model group (*E. coli* biofilm + saline), (3) vehicle control (*E. coli* biofilm + sweet almond oil), and (4) linalool treatment (25 μL/mL in sweet almond oil). After 24 h post-treatment, all rats were euthanized by an intraperitoneal injection of an overdose of sodium pentobarbital (15 mg/100 g·BW). Under aseptic conditions, the left uterine horns of rats in blank control group, model group and linalool treatment group were fixed in 2.5% glutaraldehyde for scanning electron microscopy (SEM). In contrast, the right horns of rats in all groups were washed with PBS, homogenized in 0.2% Triton X-100, serially diluted, and plated on blood agar. Bacterial loads (CFU/g tissue) were quantified following 24 h incubation at 37 °C. Inhibition ratios were calculated according to the following formula: $\text{Inhibition ratio}=[\begin{array}{l} \;(\begin{array}{l} \text{bacterial loads of the model group}-\\ \text{bacterial loads of the experimental group} \end{array})\\ /\text{bacterial loads of the}\;\;\text{model group}\; \end{array}]\times 100\%.$

### 2.7 SEM analysis of *Escherichia coli* biofilms

Biofilm structural analysis was conducted using SEM following the protocol described by Kang et al. (34). Overnight cultures of *E. coli* D5 were diluted to 3 × 10^7^ CFU/mL in LB medium and aseptically transferred (500 μL/well) onto coverslips placed in 24-well plates. Linalool was co-administered at concentrations of 0, 4, and 8 μL/mL for *E. coli* D5 (500 μL/well). Subsequently, the plates were incubated at 26 °C for 24 h under static conditions.

To analyze the effects of linalool on bacterial flagella and fimbriae, *E. coli* D5 was exposed to 1 or 2 μL/mL linalool for 30 h, while the control group received no treatment. Cells were then washed three times with PBS (pH 7.4), fixed with 2.5% glutaraldehyde for 2 h, dehydrated through a graded ethanol series (30% ~ 100%, 15 min per step), subjected to critical point drying, sputter-coated with gold, and imaged by SEM (JSM-5600, JEOL, Japan).

Uterine tissues preserved in 2.5% glutaraldehyde were rinsed twice with ultrapure water rinsing (5 min each), dehydrated through a gradient ethanol series (30% ~ 100%, 10 min per step), mounted using conductive adhesive, coated with gold-sputtered, and SEM evaluation.

### 2.8 Real-time quantitative polymerase chain reaction (RT-qPCR)

*Escherichia coli* D5 cultures exposed to linalool (0, 1, and 2 μL/mL) were incubated at 26 °C for 24 h, following the procedure outlined in Section 2.2. Biofilms were ultrasonicated for 1 min, thoroughly mixed, and centrifuged at 8,000 × *g* for 10 min. The resulting pellets were washed three times with sterile PBS, and total RNA was extracted using the OMEGA bacterial RNA kit.

First-strand complementary DNA synthesis was performed using the PrimeScript™ reverse transcriptase kit. RT-qPCR analysis was performed on an Applied Biosystems QuantStudio 5 system with TB Green™ Premix Ex Taq™ II following the cycling conditions: 95 °C for 30 s (1 cycle), 95 °C for 5 s (40 cycles), and 60 °C for 30 s. Primers for target genes (*fliA, fliG, fliM, motA, fimH, csgA,* and *csgD*) and the reference gene (16S rRNA) (Table 1) were designed and synthesized by Sangon Biotech (Shanghai). Gene expression fold changes were calculated using the 2^–ΔΔCt^ method.

**Table 1:** Primer sequences for target and reference genes.

Genes	Primer Sequences (5′–3′)	Gene size amplified (bp)
*fliA*	*fliA*-F: TTAGGGATCGATATTGCCGATT *fliA*-R: CGTAGGAGAAGAGCTGGCTGTT	70
*fliG*	*fliG*-F: GAGCTGACCGAAGTACTGAATG	120
	*fliG*-R: GGCTTCTTCCTGCTGAGTTT	
*fliM*	*fliM*-F: CAACCTGACCGGCGAATTTA	115
	*fliM*-R: GCGCCAGTTCTGATCTTCATTA	
*motA*	*motA*-F: TTGGAGCACTCTATCAACCCG	198
	*motA*-R: AGCGAAAACATCCCCATCTG	
*fimH*	*fimH*-F: CGTGCTTATTTTGCGACAGA *fimH*-R: AGGAATTGGCACTGAACCAG	166
*csgA*	*csgA*-F: GGTAACAGCGCTACTCTTGAT	119
	*csgA*-R: CGTTGACGGAGGAGTTAGATG	
*csgD*	*csgD*-F: AATCGCTGGCAATTACAGG	96
	*csgD*-R: CCGCTTCCATCATATCCAG	
*16S*	*16S-*F: CTGGAACTGAGACACGGTCC *16S-*R: GGTGCTTCTTCTGCGGGTAA	188

### 2.9 Data analysis

Statistical analyses were conducted using the Statistical Package for the Social Sciences software (version 25.0). Data are presented as mean ± standard deviation (SD). Group differences were evaluated using one-way ANOVA followed by Tukey’s test. Statistical significance was defined as *p* < 0.05, while *p* < 0.01 was considered highly significant.

## 3 Results

### 3.1 Inhibitory effects of linalool on *Escherichia coli* biofilm formation

**Figure 1:**
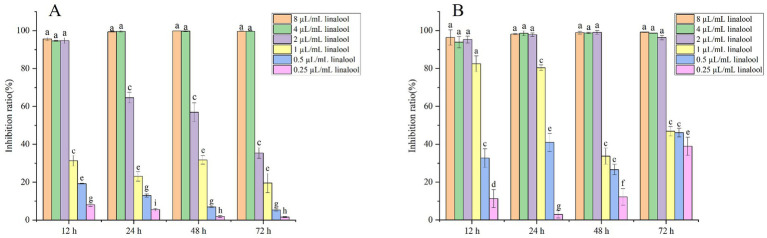
The inhibitory effect of linalool on *E. coli* biofilm: **(A)** *E. coli* D5; **(B)** *E. coli* ATCC25922. Data manifest the mean ± SD. Significant differences are indicated by different letters (*p* < 0.05 for adjacent letters; *p* < 0.01 for non-adjacent letters). *n* = 3.

The antibiofilm activity of linalool against *E. coli* D5 and ATCC 25922 was evaluated using crystal violet staining (Figure 1). *E. coli* D5 biofilms co-cultured with linalool (0.25, 0.5, 1, 2, 4, and 8 μL/mL) for 12 ~ 72 h exhibited concentration- and time-dependent inhibition (Figure 1A). At 12 h, linalool at 2 ~ 8 μL/mL achieved 90% ~ 95% inhibition. Prolonged exposure (24 ~ 72 h) with 4 ~ 8 μL/mL linalool sustained inhibition above 95%, whereas 2 μL/mL demonstrated an efficacy of less than 70%. For the ATCC 25922 strain (Figure 1B), 2 μL/mL linalool consistently inhibited biofilm formation by more than 95% across all time points. In contrast, 1 μL/mL linalool exhibited suppression (82.53% at 12 h and 80.05% at 24 h), but its effectiveness notably declined at 48 h (33.74%) and 72 h (46.88%). These findings indicated that 2 μL/mL linalool effectively inhibits biofilm formation in weak biofilm producers (ATCC 25922) and early-stage biofilm (12 h) in strong biofilm formers (D5). In contrast, higher concentrations (4 ~ 8 μL/mL) ensure sustained inhibition regardless of biofilm maturity.

**Figure 2:**
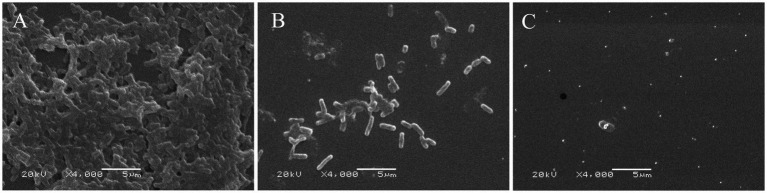
Scanning electron microscopy of *E. coli* biofilm treated by linalool for 24 h: **(A)** 0 μL/mL linalool against *E. coli* D5; **(B)** 2 μL/mL linalool against *E. coli* D5; **(C)** 4 μL/mL linalool against *E. coli* D5. The scale bars are 5 μm, *n* = 3.

SEM analysis demonstrated structural disruption of 24-h biofilms following linalool treatment (Figure 2). Untreated *E. coli* D5 exhibited dense, multilayered architectures (Figure 2A). Exposure to 2 μL/mL linalool resulted in significant biofilm thinning and bacterial reduction (Figure 2B), while complete structural collapse was observed at 4 μL/mL, resulting in only fragmented cells (Figure 2C). These findings highlight linalool’s ability to destabilize biofilm integrity through biomass reduction and ultrastructural degradation.

### 3.2 Linalool inhibits motility of *Escherichia coli* D5

**Figure 3:**
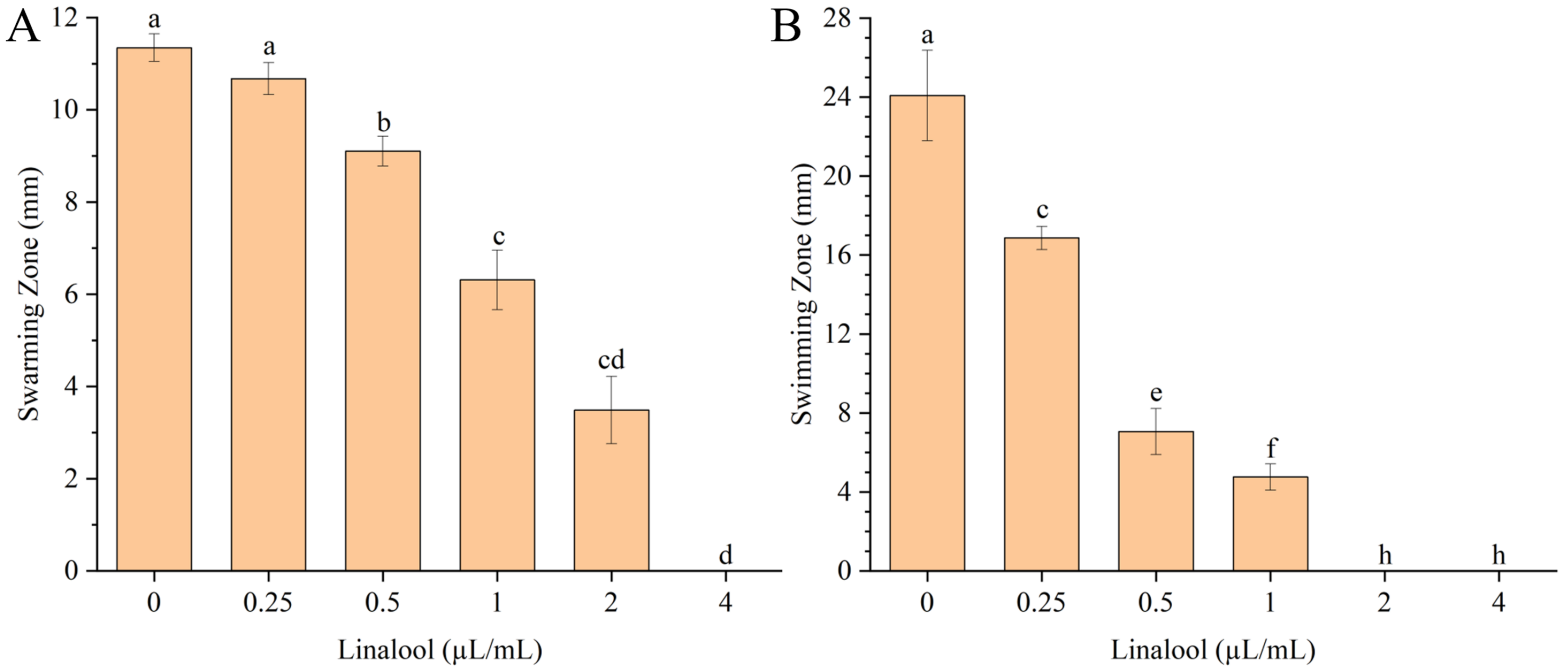
Effects of linalool on motilities of *E. coli* D5: **(A)** Swarming motility; **(B)** Swimming motility. Data manifest the mean ± SD. Significant differences are indicated by different letters (*p* < 0.05 for adjacent letters; *p* < 0.01 for non-adjacent letters). *n* = 3.

The inhibitory effects of linalool on *E. coli* D5 motility were assessed using swarming and swimming assays (Figure 3). Swarming motility analysis (Figure 3A) revealed significant inhibition (*p* < 0.05) at 0.5 μL/mL linalool, with a progressive reduction in diameter at higher concentrations. Complete suppression of swarming was observed at 4 μL/mL linalool, indicating a dose-dependent inhibitory effect within the 0.25 ~ 4 μL/mL range.

Similarly, swimming motility assays (Figure 3B) exhibited significant suppression (*p* < 0.01) at 0.25 μL/mL linalool, with complete inhibition occurring at 2 μL/mL. A concentration-dependent inhibitor effect was observed across the 0.25 ~ 2 μL/mL linalool. These results confirmed linalool’s effectively suppressed swarming and swimming motilities in *E. coli* D5 in a dose-dependent manner, with swarming inhibition requiring higher concentrations for complete suppression.

### 3.3 Linalool suppresses flagella and fimbriae formation in *Escherichia coli* D5 biofilms

**Figure 4:**
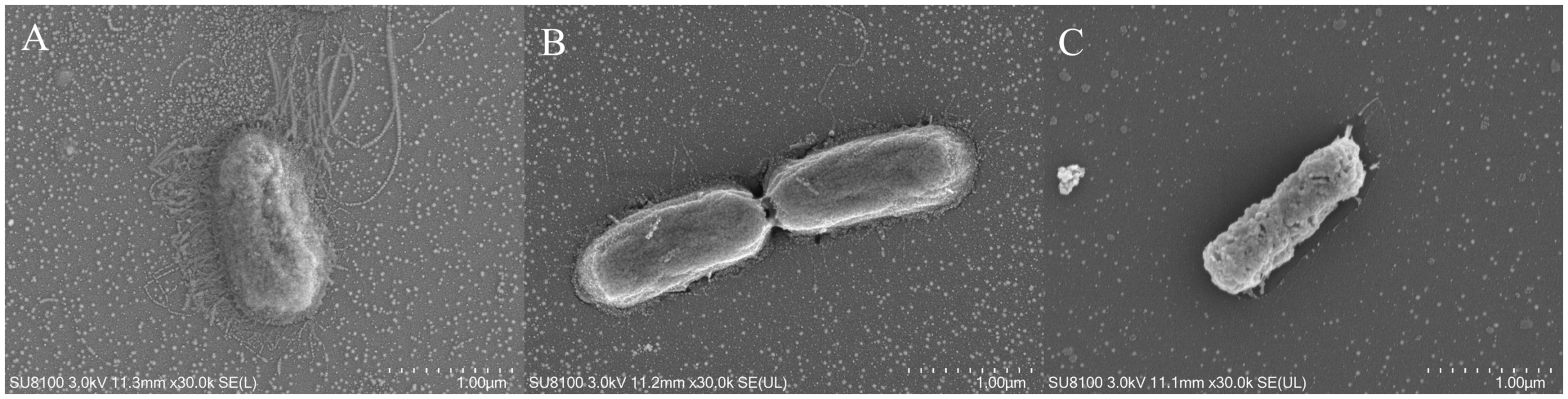
Scanning electron microscopy observation of the effect of linalool on bacterial flagella and fimbriae during the formation of biofilm in *E. coli* D5: **(A)** Control group; **(B)** 1 μL/mL linalool; **(C)** 2 μL/mL linalool. The scale bars are 1 μm, *n* = 3.

The impact of linalool on flagella and fimbriae formation during biofilm development was assessed by treating *E. coli* D5 with linalool (0 ~ 2 μL/mL) for 30 h, followed by SEM analysis (Figure 4). Untreated controls exhibited abundant flagella and fimbriae, essential for bacterial motility and adhesion (Figure 4A). Exposure to 1 μL/mL linalool significantly reduced flagellar density and fimbriae length (Figure 4B). At 2 μL/mL linalool, complete structural elimination of flagella and fimbriae was observed, accompanied by cellular shrinkage and surface pitting (Figure 4C), indicating concentration-dependent ultrastructural disruption.

**Figure 5:**
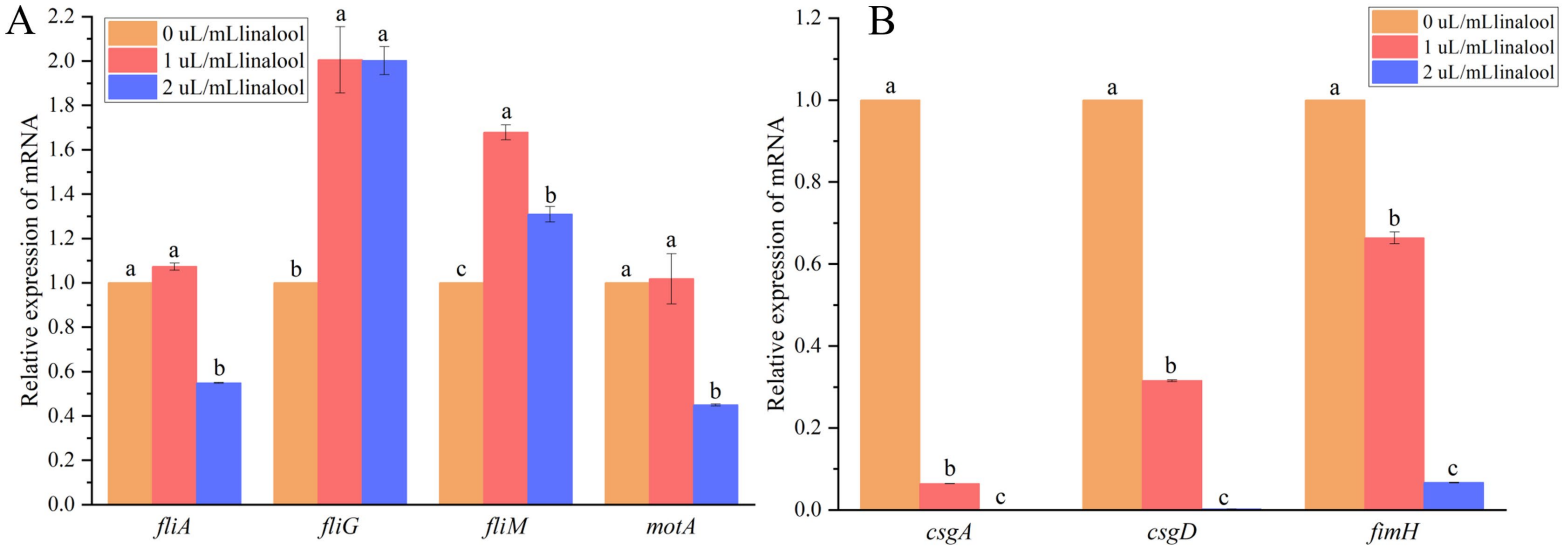
The effect of linalool on the relative expression levels of flagella and fimbriae mRNA during the formation of biofilm in *E. coli* D5: **(A)** Flagella related genes; **(B)** Fimbriae related genes. Data manifest the mean ± SD. Significant differences are indicated by different letters (*p* < 0.05 for adjacent letters; *p* < 0.01 for non-adjacent letters). *n* = 4.

RT-qPCR analysis (Figure 5) demonstrated dose-dependent transcriptional regulation of key motility- and adhesion-related genes in *E. coli* D5 following linalool treatment. Flagellar rotor genes (*fliG* and *fliM*) were upregulated (*p* < 0.05) at 1 and 2 μL/mL linalool (Figure 5A). In contrast, the motility motor gene (*motA*) and the flagellar regulatory gene (*fliA*) were significantly downregulated at 2 μL/mL (*p* < 0.05). For adhesion-related genes (Figure 5B), linalool (1 ~ 2 μL/mL) downregulated the expression of curli-associated genes (*csgA* and *csgD*) and type I fimbriae gene (*fimH*) (*p* < 0.05). These transcriptional alterations align with the observed impairment in motility and adhesion, suggesting that linalool disrupts biofilm formation through dual inhibiting structural appendages and their regulatory pathways.

### 3.4 Linalool inhibits *Escherichia coli* D5 adhesion to bovine endometrial epithelial cells

**Figure 6:**
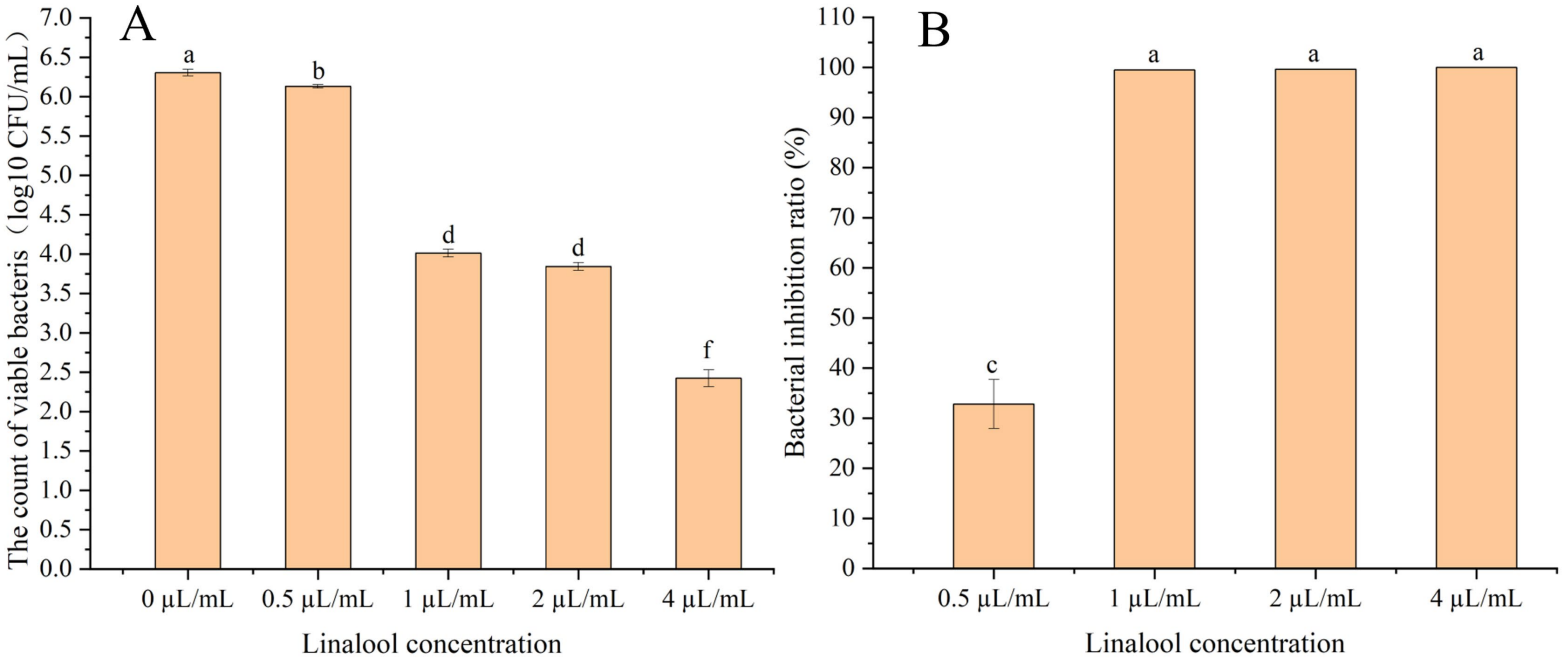
Effect of linalool on adhesion of *E. coli* D5 to endometrial epithelial cells of dairy cows: **(A)** The count of viable bacteria; **(B)** Bacterial inhibition ratio. Data manifest the mean ± SD. Significant differences are indicated by different letters (*p* < 0.05 for adjacent letters; *p* < 0.01 for non-adjacent letters). *n* = 3.

Linalool significantly inhibited *E. coli* D5 adhesion to bovine endometrial epithelial cells in a concentration-dependent manner, as determined by viable count assays (Figure 6). Linalool at 0.5 μL/mL reduced adherent bacteria to 67.21% of the control level (*p* < 0.05). A marked suppression was observed at concentrations ≥ 1 μL/mL, with bacterial loads reduced to 5.13‰ (99.49% inhibition, *p* < 0.01), 3.46‰ (*p* < 0.01), and 0.13‰ (99.99% inhibition, *p* < 0.01) following treatment with 1, 2, and 4 μL/mL, respectively. Statistical comparison indicated significant differences between 0.5 and 1 μL/mL (*p* < 0.01) as well as 2 and 4 μL/mL (*p* < 0.01). In contrast, a statistically non-significant difference was observed between 1 and 2 μL/mL (*p* > 0.05) (Figures 6A,B).

**Figure 7:**
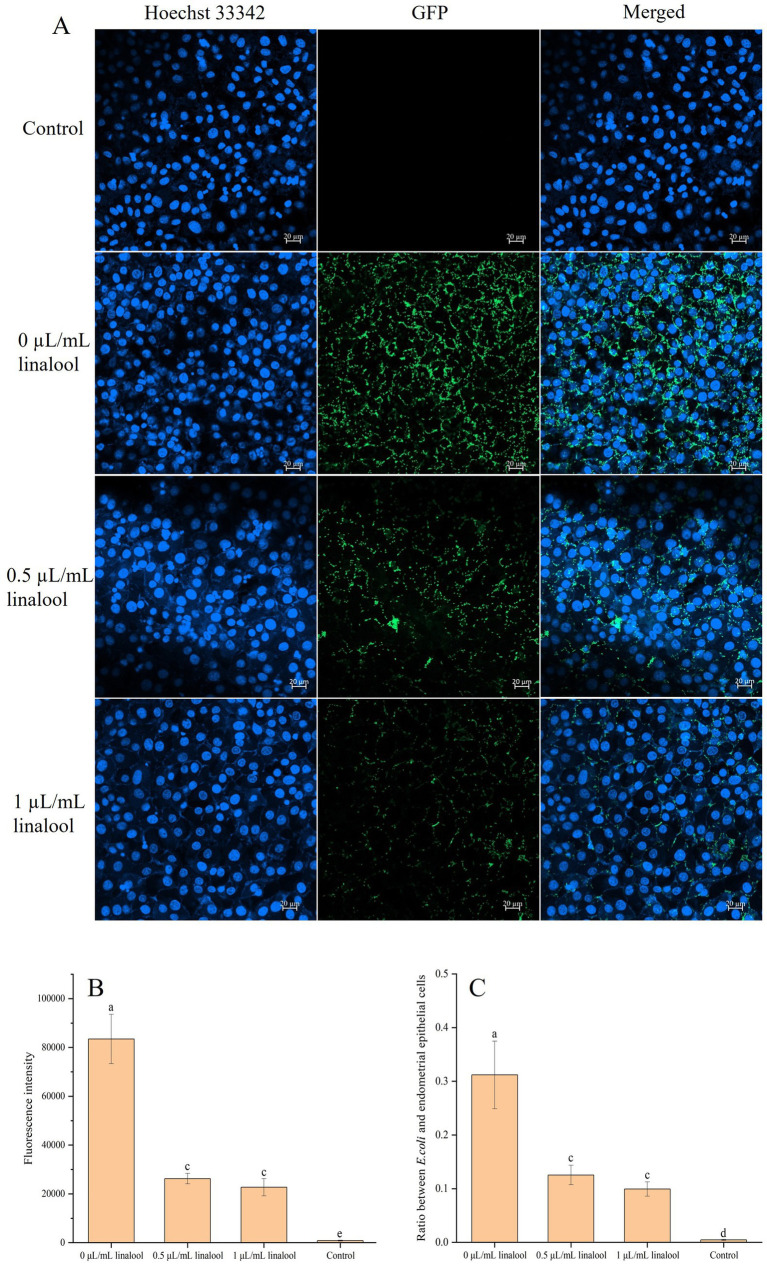
The inhibiting effect of linalool on *E. coli* D5 adhered on endometrial epithelial cells of dairy cows: **(A)** CLSM images; **(B)** Fluorescence intensity of *E. coli* D5; **(C)** Ratio between *E. coli* D5 and endometrial epithelial cells. Endometrial epithelial cells of dairy cows, blue; *E. coli* D5, green. The scale bars are 20 μm, *n* = 3. Data manifest the mean ± SD. Significant differences are indicated by different letters (*p* < 0.05 for adjacent letters; *p* < 0.01 for non-adjacent letters).

CLSM analysis further supported these findings (Figure 7). Untreated controls exhibited dense GFP-tagged *E. coli* D5 adhesion (green fluorescence) surrounding Hoechst 33342-stained nuclei of endometrial epithelial cells of dairy cows (blue fluorescence) (Figure 7A). Linalool treatment (0.5 ~ 1 μL/mL) progressively reduced bacterial fluorescence intensity and ratios between *E. coli* D5 and endometrial epithelial cells (Figures 7B,C), confirming the suppression of linalool on bacterial adhesion.

### 3.5 Linalool inhibits Intrauterine *Escherichia coli* D5 adhesion in a rat model

**Figure 8:**
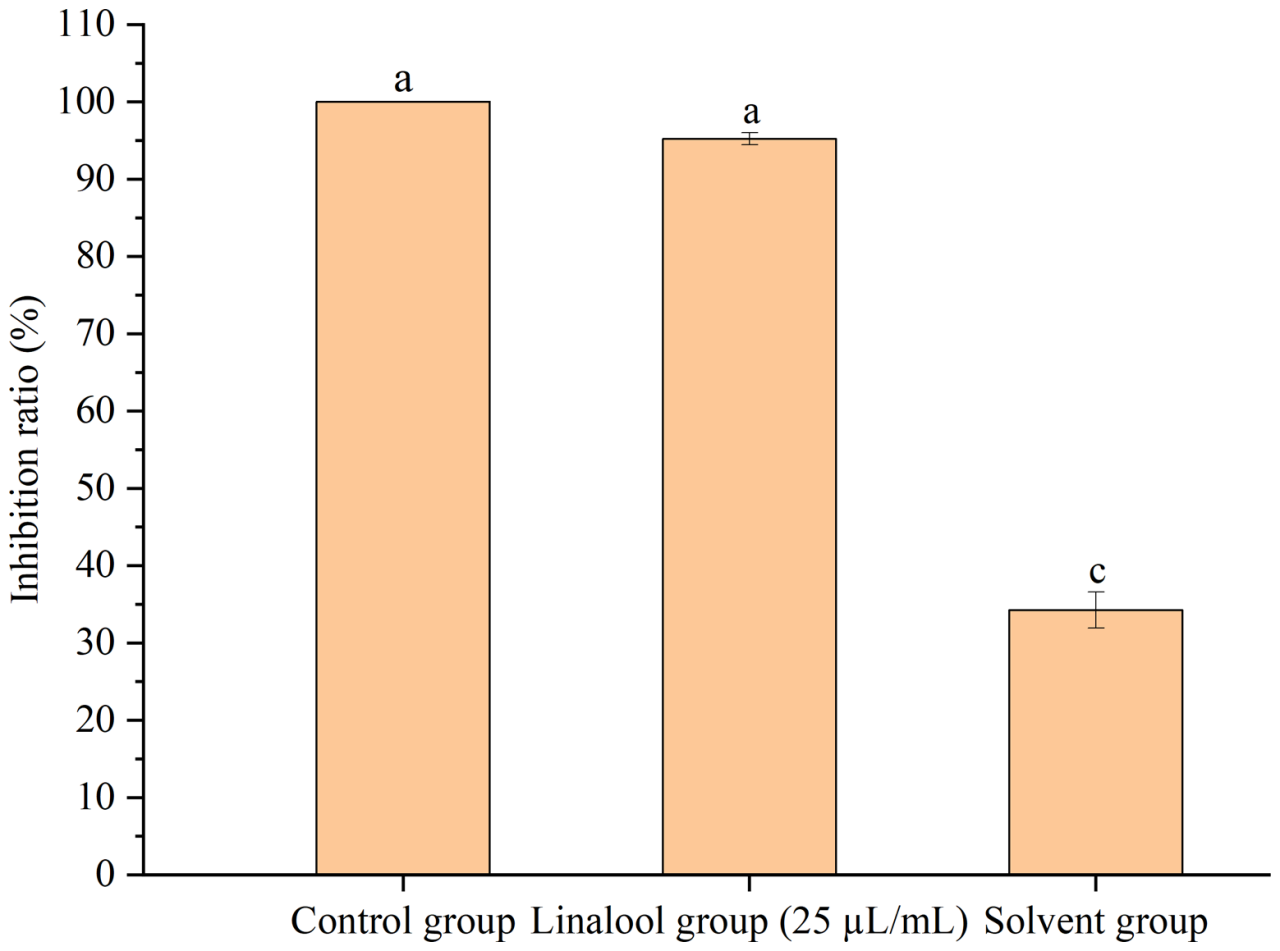
Inhibitory effect of linalool on the adhesion of *E. coli* D5 in rat uterus *in vivo* (*n* = 6) Notes: Data manifest the mean ± SD. Significant differences are indicated by different letters (*p* < 0.05 for adjacent letters; *p* < 0.01 for non-adjacent letters).

The *in vivo* anti-adhesion efficacy of linalool was evaluated using a rat intrauterine infection model. *E. coli* D5 biofilms (10^10^ CFU/mL) were introduced into the uterine cavities, followed by 24-h treatment with 25 μL/mL linalool in a sweet almond oil carrier. Viable count assays demonstrated a significant bacterial load reduction, with linalool decreasing bacterial colonization to 4.77% of the model group (*p* < 0.01), whereas vehicle controls (sweet almond oil) retained 65.75% residual bacteria (Figure 8). Blank controls exhibited no detectable bacterial presence.

**Figure 9:**
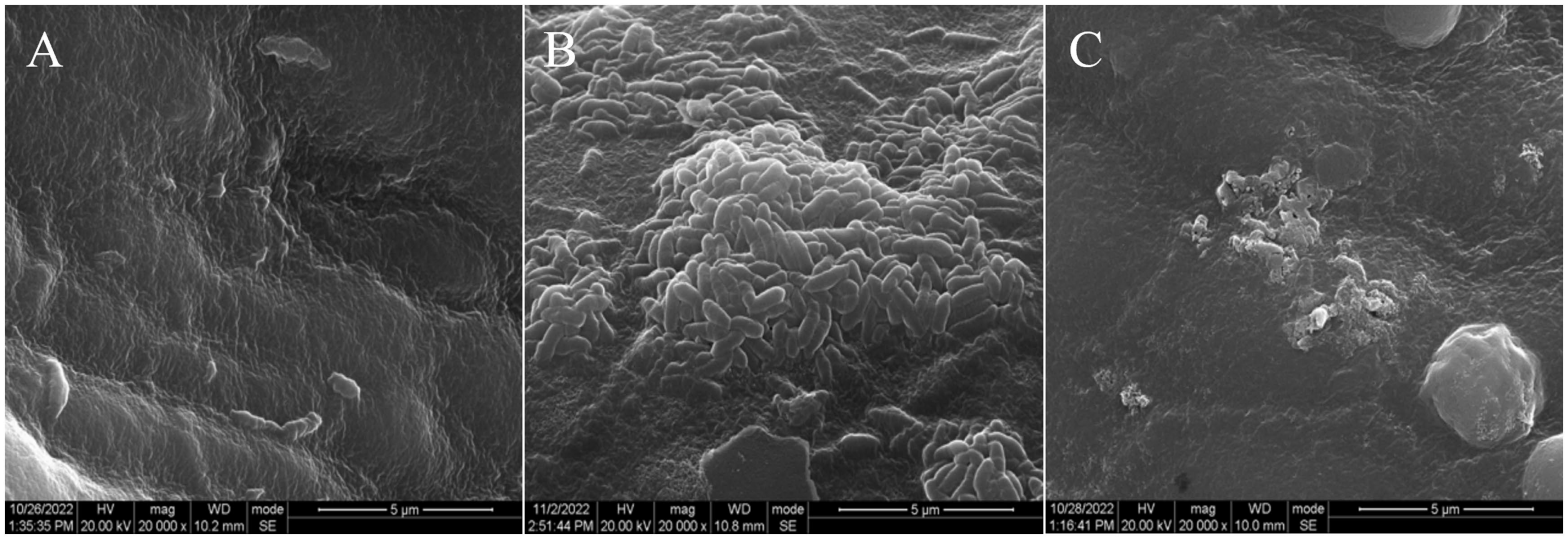
Scanning electron microscope image of *E. coli* in uterus of rat: **(A)** Blank control group; **(B)** Model group; **(C)** 25 μL/mL linalool group. The scale bars are 5 μm, *n* = 3.

SEM analysis (Figure 9) revealed dense biofilm-like structures with stacked bacilli in the model group uteri. In contrast, linalool-treated uteri exhibited fragmented bacterial remnants without intact biofilms. These findings confirm the potent intrauterine anti-adhesion activity of linalool, exceeding vehicle efficacy by over 60% (*p* < 0.01), highlighting therapeutic potential against biofilm-associated uterine infections.

## 4 Discussion

Linalool, a monoterpenoid alcohol widely utilized in cosmetics, detergents, and food additives, exhibits diverse biological activities, including antimicrobial and anti-inflammatory properties (20). Despite its extensive applications, its antibiofilm mechanisms against *E. coli* remain unclear. Lahiri et al. (9) reported that linalool inhibits the protein and carbohydrate components of biofilm exopolysaccharide and quorum-sensing proteins in *P. aeruginosa*. Similarly, Shen et al. (21) demonstrated that linalool suppresses cell motility and reduces the production of exopolysaccharides and biofilm matrix proteins in *Bacillus amyloliquefaciens*. While these findings highlight the multifaceted antibiofilm effects of linalool, the precise mechanisms underlying its inhibition of bacterial motility and its impact on bacterial adhesion remain unclear. The present study elucidates a novel dual-action mechanism by which linalool disrupts *E. coli* D5 biofilms through motility suppression and adhesion blockade.

Biofilm initiation relies on surface attachment facilitated by flagellar motility (22). The flagellar apparatus, consisting of FliG/FliM rotor proteins and MotA/MotB stator complexes (12), enables both swimming (individual propulsion) and swarming (collective migration) (23). Treatment with 2 μL/mL linalool significantly downregulated *fliA* (flagellar transcriptional regulator) and *motA* (motor protein gene) while upregulating *fliG* and *fliM* (Figure 5A). This contradictory gene expression pattern, where structural components (*fliG* and *fliM*) enhanced while functional regulators (*fliA* and *motA*) were suppressed, possibly disrupts flagellar assembly kinetics. SEM analysis confirmed a dose-dependent reduction in flagellar structures (Figure 4C), corresponding with impaired swarming and swimming motility (Figure 3). This dual-phase interference suggests that linalool destabilizes flagellar integrity by disrupting the coordination between structural biosynthesis and functional maturation.

Fimbriae-mediated adhesion plays a crucial role in stabilizing surface colonization. Curli (*csgA* and *csgD*) and type I fimbriae (*fimH*) facilitate irreversible attachment and biofilm matrix consolidation (24, 35). Linalool treatment considerably suppressed the expression of relative genes such as *csgA*, *csgD*, and *fimH* (Figure 5B), aligning with previously reported phytochemical strategies. For example, Ginkgo extracts inhibited curli production (25), phloretin downregulated *csgAB* genes (26), and eugenol blocked *csgABDFG/fimCDH* expression (15). SEM analysis revealed fimbriae truncation at 1 μL/mL linalool (Figure 4B) and complete elimination at 2 μL/mL linalool (Figure 4C). These findings are consistent with the report demonstrating that inhibition of type I fimbriae impaired *E. coli* biofilm formation (27).

Notably, linalool significantly reduced *E. coli* D5 adhesion to biological substrates, including endometrial epithelium and uterus, which may be closely associated with decreased fimbriae. This finding aligns with a previous report by Sheng et al. (28), which demonstrated that curli fimbriae in *E. coli* O157: H7 enhance biofilm formation, epithelial cell invasion, and persistence in cattle. Additionally, prior studies have established that fimbriae abundance directly influences bacterial adherence (29, 30). Endometrial epithelial cells are the primary defensive barrier against pathogens within the maternal reproductive system. These cells detect intrauterine pathogens through Toll-like receptor-mediated recognition of bacterial components such as lipopolysaccharides, triggering pro-inflammatory cytokine production (31) and facilitating intercellular communication (32). Endometrial epithelial integrity is essential for critical reproductive functions, including embryo implantation and pregnancy maintenance. This study’s findings highlight linalool’s dual protective role: inhibiting bacterial adhesion to endometrial surfaces *in vitro* and eliminating intrauterine biofilms *in vivo*, promoting uterine health. This dual mechanism, characterized by impairment of bacterial motility and adhesion while preserving host tissue integrity, effectively disrupts biofilm formation and attenuates *E. coli* pathogenicity. However, cinnamaldehyde reduced the initial adhesion of bacteria and delayed the formation of biofilms rather than inhibiting flagellar-mediated motility (33). The specific inhibitory mechanism of linalool against the formation of *E. coli* biofilms demonstrates its potential as an anti-biofilm agent.

## 5 Conclusion

Linalool effectively combats *E. coli* D5 biofilms through a multimodal mechanism. It disrupts flagellar motility by modulating gene expression, specifically suppressing *fliA* and *motA* while upregulating *fliG* and *fliM*. Additionally, it inhibits fimbriae-mediated adhesion by downregulating *csgA*, *csgD*, and *fimH*, preserves endometrial epithelial integrity, reduced bacterial adhesion by more than 99.99% *in vitro*, and eliminated 95.23% of intrauterine biofilms *in vivo*. These findings underscore linalool’s potential as a natural therapeutic agent against biofilm-associated infections, particularly within the urogenital tract, warranting further investigation into its synergistic effects with antibiotics and efficacy against multidrug-resistant strains.

## Data Availability

The datasets presented in this study can be found in online repositories. The names of the repository/repositories and accession number(s) can be found in the article/Supplementary material.
